# Challenges to Reporting Medical Device-Associated Adverse Events: Perspectives of Various Stakeholders

**DOI:** 10.7759/cureus.104272

**Published:** 2026-02-26

**Authors:** Devu Raju, Princy L Palatty, Abhishek Anil Nair, Maheshkumar D, Sai Bala M, Radhika Mohandas, Vimal Vijayan

**Affiliations:** 1 Pharmacology, Amrita Institute of Medical Sciences and Research Centre, Amrita Vishwa Vidyapeetham, Kochi, IND; 2 Pharmacy, Manipal College of Pharmaceutical Sciences, Udupi, IND; 3 Nursing Services, Amrita Institute of Medical Sciences and Research Centre, Amrita Vishwa Vidyapeetham, Kochi, IND; 4 Nursing Quality Division, Amrita Institute of Medical Sciences and Research Centre, Amrita Vishwa Vidyapeetham, Kochi, IND; 5 Emergency Medicine, Amrita Institute of Medical Sciences and Research Centre, Amrita Vishwa Vidyapeetham, Kochi, IND

**Keywords:** awareness, challenges, materiovigilance, medical device-related adverse events, reporting

## Abstract

Background

The effective operation of a medical device surveillance system relies heavily on healthcare professionals and other stakeholders voluntarily reporting adverse events related to medical devices. Despite measures to increase awareness among stakeholders and ensure user-friendly reporting procedures, the materiovigilance (MV) program is yet to pick up pace. Studies on knowledge and reporting of medical device-associated adverse events have been carried out in other countries, while in India, there has been limited investigation into this. This study aimed to understand the readiness of healthcare professionals in reporting adverse events related to medical devices.

Objectives

The objectives of this study were to identify hurdles to reporting medical device-related adverse events among healthcare workers, determine the awareness of MV among healthcare professionals, and resolve the challenges faced.

Methodology

This qualitative study used purposive sampling to conduct focus group discussions with selected healthcare workforce, including clinicians, nursing staff, pharmacists, and administrators from a tertiary care teaching hospital. Following Institutional Ethics Committee or IEC approval, we obtained informed consent from all participants before commencing the study. Each group consisted of six to eight participants to ensure diverse input across different levels of seniority and policy-making. A semi-structured framework was used to evaluate awareness, reporting experiences, and strategies to overcome barriers in MV. The discussions were recorded and transcribed. The transcribed data were thematically analyzed using inductive qualitative coding to develop subthemes and themes.

Results

The four themes elicited from the codes and subthemes were knowledge and awareness, hurdles in reporting, socioeconomic and logistical factors, importance of reporting, and resolutions in MV. The study identified a significant disparity in knowledge. Participation was further restrained by psychological and logistical hurdles, including a fear of professional blame and the burden of documentation. The important phrases that came to the fore, quoted verbatim, were as follows: (1) “if we report, they might get into trouble” and (2) “is there a Google form; the writing process is too much.” Challenges such as a lack of institutional support, vendor-related problems, and device quality issues were found to directly impact patient safety. To mitigate these risks, participants emphasized the need for structured resolutions, including feedback and appreciation, inter-departmental collaboration, and enhanced vendor accountability.

Conclusion

Effective MV is crucial for patient safety and device quality, yet it requires robust training and a culture of feedback to encourage consistent reporting. Implementing accessible reporting tools such as digital forms or dedicated registers allows monitoring centers to streamline data collection and receive better support from healthcare staff. A no-blame environment is essential to encourage honest reporting.

## Introduction

Materiovigilance (MV) is “the coordinated system of identification, collection, reporting, and analysis of any untoward occurrences associated with the use of medical devices. It safeguards patient safety by preventing the recurrence of medical device-associated adverse events (MDAEs) and by taking necessary actions, such as field safety corrections or device recalls [1]. MV also aims to aid in regulatory decision-making and recommendations on the safe use of medical devices, thereby protecting patient health by preventing recurrences due to MDAEs [2]. While postmarketing surveillance of the devices is less developed compared with medicines, the International Medical Device Regulators Forum (IMDRF), comprising 10 countries, such as the USA (MedWatch), Japan, Europe (MDA), New Zealand (MedSafe), China, and South Korea, was set up in 2011 to introduce the concept and implementation of the MV program to monitor MDAEs and to harmonize international medical device regulation via MV [3,4]. This made it easier for the developing countries such as India to later launch their own program, the MV Programme of India (MVPI), which was launched on July 6, 2015 at the IPC, Ghaziabad by the Drugs Controller General India (DCGI), seeking to track adverse events, generate safety data, raise stakeholder awareness, and recommend best practices to enhance patient safety [3,5].

However, the effective operation of a medical device surveillance system relies heavily on healthcare professionals and other stakeholders voluntarily reporting adverse events related to medical devices [1]. Studies on inadequate knowledge and underreporting of MDAEs have been carried out in other countries, while in India, there has been limited investigation into the knowledge, attitudes, and practices of healthcare professionals concerning MV [1]. The need for a qualitative, stakeholder-focused exploration in India about MV is implied by the objectives of the MV program to create awareness and improve patient safety [5]. Therefore, in this study, we plan to explore the opinions and beliefs of healthcare professionals toward MV. We also try to find the reluctance in reporting MDAEs, with a view to understanding the attitudes and device methods to offset them.

## Materials and methods

Study design and ethics

Upon the phenomenological basis, a focus group discussion (FGD) was the method used to elicit in-depth, collective insights on MDAEs, which was conducted after obtaining Institutional Ethics Committee (IEC) approval with reference number IEC-AIMS-2024-PHARMACO-184. 

Participant selection and recruitment

Participants were selected using a purposive sampling technique to ensure the recruitment of a dense sample of healthcare professionals, involving seven groups of doctors, nurses, pharmacists, and hospital administrators (6-10 participants per group), from Amrita Institute of Medical Sciences and Research Centre, Kochi, with direct experience in the core content. They are Indian residents aged between 20 and 50 years, with proficiency in English and/or Malayalam. This specific demographic and professional profile ensured that the participants possessed the necessary expertise to provide meaningful data regarding the study's objectives. Comfortable seating and refreshments were made available to all participants, and informed consent was obtained, which highlights that their involvement was optional and their answers would be kept confidential.

Sample size and information power

The sample size of this study (n=60) was determined based on the principle of information power (Ip), which states that in a qualitative study, the more relevant information a sample holds, the fewer participants are required [6]. Given that the study's aim was specific, with all participants possessing highly relevant professional expertise, a moderator-guided discussion using a structured agenda with five preamble questions and a systematic thematic analysis, a high level of Ip was achieved.

To minimize bias, the moderator refrained from expressing opinions and used open-ended, non-leading prompts throughout the discussion. The five preamble questions that were developed based on the previous MV studies helped to direct the discussion on participants' experiences regarding medical device safety (Table 1). 

**Table 1:** Preamble questions and duration of the focus group discussion MVPI, Materiovigilance Programme of India.

No.	Preamble questions	Duration
1	Are you aware of MVPI?	5 minutes
2	Have you reported any medical device-related adverse events?	5 minutes
3	What are the particular medical device-related adverse events that you encounter in your daily work?	5 minutes
4	What are the difficulties that you commonly encounter in your daily work?	20 minutes
5	How can we improve the quality of adverse event reporting?	20 minutes

**Figure 1:**
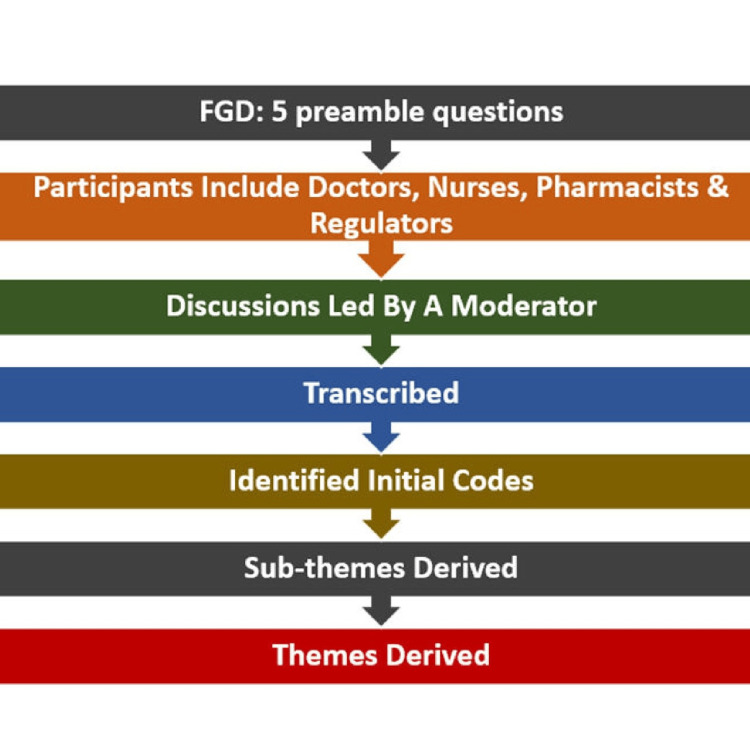
Methodological framework for data collection through focus group discussions and subsequent derivation of thematic insights FGD, focus group discussion.

The data collection was continued until thematic saturation was reached, with no new themes emerging from subsequent discussions. Each session lasted for about one hour, which was recorded and later transcribed (Figure 1).

Data analysis

Consistent with the methodological framework, the data were systematically analyzed through a step-by-step thematic process. This approach was used to ensure credibility as described below.

Transcription

Audio recordings from the seven groups were transcribed; verbatims were noted by multiple investigators and cross-checked for linguistic accuracy. 

Initial Coding

An open coding process was used to identify recurring concepts and significant statements.

Sub-theme Derivation

Initial codes were categorized into sub-themes based on conceptual patterns.

Thematic Derivation

The final themes were derived to capture the participants’ shared experiences.

**Figure 2:**
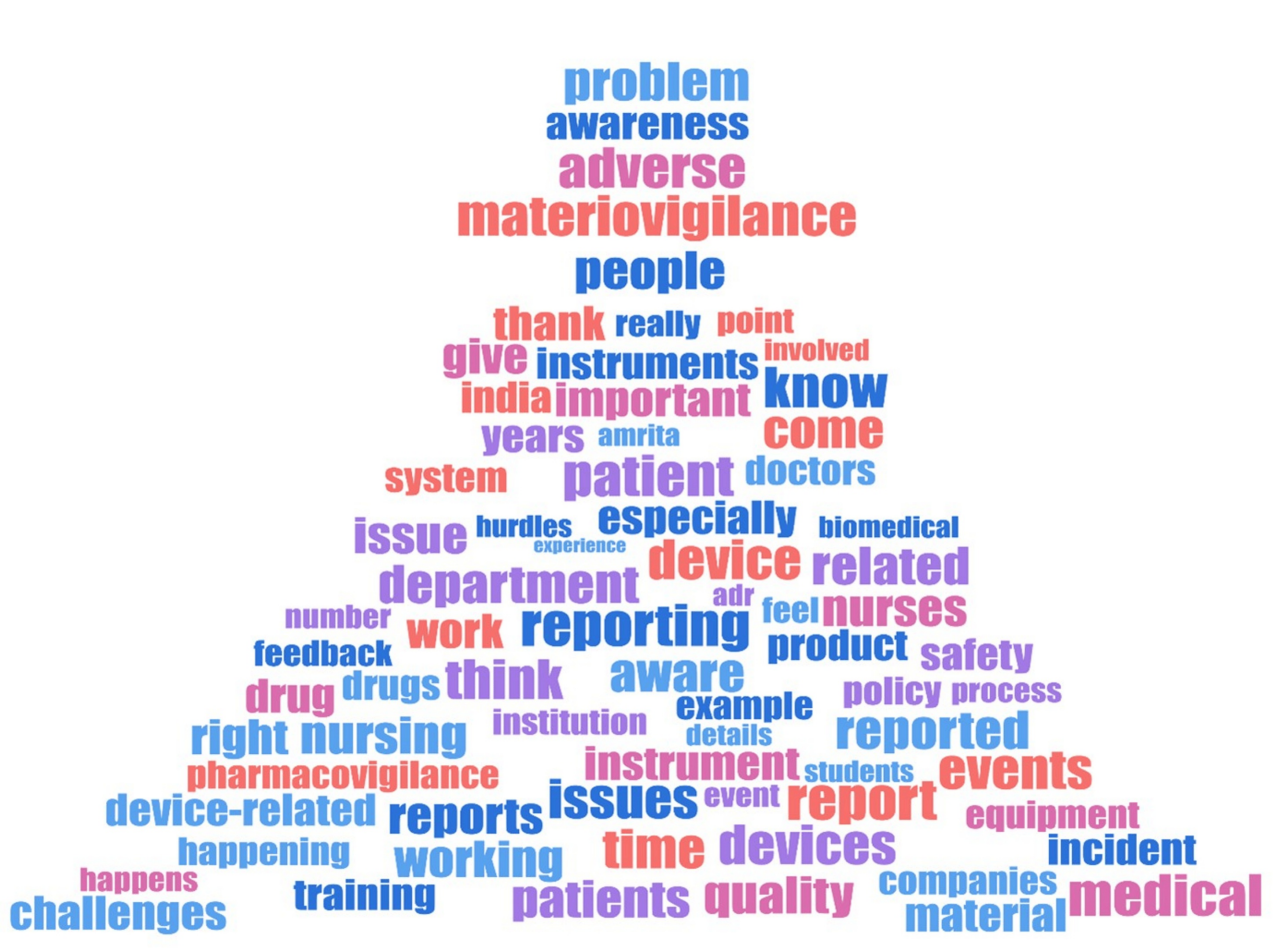
Word cloud illustrating the most frequent terms and concepts identified during focus group discussions on materiovigilance

A word cloud was generated using the trial version of MAXQDA 2022 software (VERBI Software GmbH, Berlin, Germany), which showed the selected words that have cropped up in the FGDs (Figure 2). 

## Results

Sociodemographic characteristics

Seven sessions of FGD (two sessions with doctors, two sessions with nurses, two sessions with pharmacists, and one session with administrators) were conducted, comprising 60 participants based on their professional distribution, gender breakdown within each profession, and age distribution across those professions (Table 2). There were 24 males and 36 females, with a mean age of 35 ± 15 years.

**Table 2:** Participant gender distribution across professional groups

Stakeholder group (n)	Male (n, %)	Female (n, %)
Doctors (17)	11 (64.7%)	6 (35.3%)
Nurses (21)	0 (0.0%)	21 (100.0%)
Pharmacists (15)	9 (60.0%)	6 (40.0%)
Administrators (7)	4 (57.1%)	3 (42.9%)
Total (60)	24 (40.0%)	36 (60.0%)

Thematic analysis of reporting challenges and proposed solutions

**Figure 3:**
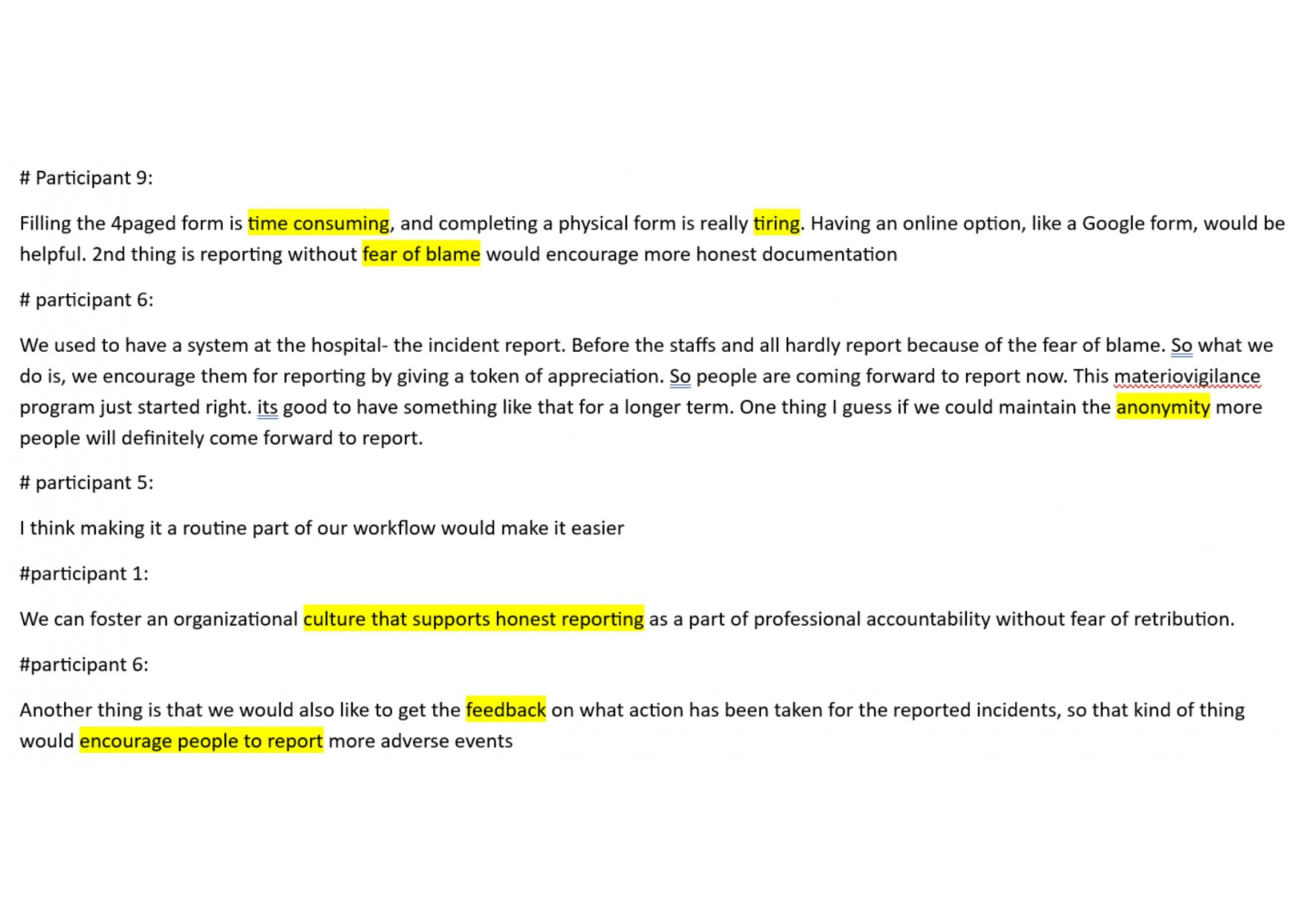
Illustration of the identification of key codes from the transcript (administrator group of participants)

The initial stage of analysis involved identifying important phrases from the transcribed FGD as codes and grouping them into similar ideas (Figure 3). Some of the important phrases that came to fore, quoted verbatim, were as follows: (1) “if we report, they might get in trouble,” (2) “I was not aware of materiovigilance,” (3) “I'm also not knowing that there was some functional thing like this for device related events,” (4) “it's a new instrument. Who will take the blame? They are afraid of the blame, so they're not going to report or take the blame,” and (5) “they often prioritize profits over patient safety and delay responses to complaints until the hospital's stock of the faulty item is depleted.”

Thematic analysis of the FGDs revealed multifaceted challenges influencing the reporting of MDAEs, leading to the identification of emergent themes and associated sub-themes, as summarized in Table 3.

**Table 3:** Major themes and associated sub-themes identified through thematic analysis

Themes	Sub-themes
Knowledge and awareness gaps	Lack of awareness and uncertainty about reportable incidents
Psychological and procedural barriers	Fear of blame and consequences, time constraints, and documentation burden
Socioeconomic and logistical factors	Economic constraints and device-related quality concerns, inadequate institutional support, delays in manufacturer and regulatory response, and vendor accountability (blaming staff and prioritizing profits)
Resolutions	Sensitization and training programs, assurance of anonymity and confidentiality, streamlined reporting process, quality improvement measures, interdepartmental collaboration, and vendor compliance management

Category-wise view

Though common themes emerged across groups, variations were observed in how different stakeholders prioritized specific reporting challenges and solutions (Table 4). Each group's unique role provided a different lens on systemic failures. 

**Table 4:** Stakeholder-specific perspectives on reporting challenges and the proposed strategies

Healthcare professionals	Key challenges identified	Suggested solutions
Doctors	Limited sensitization, blame culture, and cumbersome reporting procedures	Establishment of a no-blame culture, providing user-friendly online reporting platforms, and feedback and recognition mechanisms
Nurses	Knowledge disparities, time constraints, and logistical barriers in retrieving device details (batch numbers from discarded packaging)	Comprehensive training for the healthcare workers, including technicians, biomedical engineers, and respiratory therapists, and integrating the reporting system into the existing workflow via the hospital information system
Pharmacists	Information gap, fear and hesitancy, and inadequate documentation (as the faulty device is often discarded before details can be logged)	Promotion of psychological safety, setting up a register and a tray (for storing faulty devices before reporting), collaboration with the hospital store department (already documents faulty items for returns in a register)
Management and regulators	Fear of blame as a systemic barrier, reporting process complexity, and the range of device-specific problems (lifespan limitations, variable quality, malfunctions, and complications) pose a direct challenge to patient safety.	Simplified reporting procedure, feedback mechanisms, and institutional sensitization programs

## Discussion

In this study, a prominent finding was the disparity between awareness and the practical channels of reporting. While pharmacovigilance was a familiar concept, many doctors, junior pharmacists, and interns were unfamiliar with the term MV. Sojitra et al.'s study pointed out an overwhelming gap between awareness and actual reporting practices [7]. In contrast, physicians typically submitted device-related incidents through internal hospital reporting systems. The pharmacists reported that their training focused mainly on MV, revealing a gap in MV education. Senior nurses and administrators described an established internal reporting pathway linked to the national MV program under the Pharmacovigilance Programme of India. Studies conducted by Sivagourounadin et al. and Srinivas et al. also mention that nurses and healthcare workers generally possess sufficient knowledge and positive attitudes toward MV, but there is a significant disconnect between their understanding and reporting practices [1,8].

We could identify several significant hurdles in the way of effective MV. Fear of blame consistently tops the list of hurdles to reporting. The doctors described that in teaching institutions, where distinctions between user error and device malfunction are often unclear, reporting was frequently perceived as a mechanism for assigning responsibility rather than improving safety. This perception contributed to concerns about professional repercussions and potential financial liability. This was validated by the administrators’ group, who confirmed the stress on the staff regarding accountability for damaged devices. The pharmacists highlighted reluctance among nursing staff to report incidents that might implicate colleagues. The study by Sojitra et al. supports these findings, with nurses failing to report fearing repercussions [7]. In addition to these cultural barriers, the procedural complexity further increased these challenges. The complex four-page paper form for reporting the MDAEs was described as time-consuming and difficult to complete in busy clinical settings. Participants felt that simplifying the process and reducing documentation requirements could improve reporting rates. Sojitra et al. have made the same observations regarding their recommendations to improve the effectiveness of MV [7]. The management confirmed that the complexity of the process leads to the omission of details while filling out the form. Another obstacle noted by senior nurses and pharmacists to reporting was that in some areas, such as operating theaters and emergency units, where device packaging containing important batch details is often discarded immediately, accurate reporting is difficult at a later stage.

Socioeconomic and logistical factors also limited effective MV reporting. In the Indian setting, the reuse of certain single-use devices to reduce costs creates accountability challenges, as vendors often deny responsibility when such devices fail. Participants reported examples of poor-quality or malfunctioning devices and noted that vendors frequently attributed defects to user error. The pharmacists’ group of participants identified a critical gap between internal documentation and formal reporting. The hospital's store department logs faulty devices for returns but does not formally feed this data into the MVPI. The administrators’ group described instances where manufacturers initially denied defects but later replaced entire batches after evidence was provided, highlighting the need for stronger follow-up systems and better integration between institutional and national reporting mechanisms. These findings align with the study conducted by Shukla et al. [9].

Our findings also identified several complementary strategies to strengthen MV. A strong emphasis was placed on structured education and training programs that clarify the importance of reporting, the types of reportable incidents, and the reporting process. These findings are echoed in the studies by Shukla et al. and Sojitra et al., which have highlighted the need for regular training to instill a sense of responsibility for reporting medical device adverse events and to ensure the quality of reported data [7,9]. Doctors and administrators strongly stood for a simplified, streamlined, user-friendly reporting system, such as Google Forms (Google, Mountain View, CA) or QR codes, while nurses preferred integration within the existing hospital information system to ensure seamless workflow. They advocated improving product identification and ensuring proper documentation to enhance reporting efficiency. The pharmacists proposed maintaining a dedicated logbook and storage tray in clinical areas to document incidents and preserve faulty devices for proper reporting. Across all stakeholder groups, the need for a non-punitive reporting culture was strongly emphasized. Participants noted that consistent feedback and visible action following reports would encourage continued engagement. A study conducted by Shukla et al. stressed the importance of acknowledging that those who report data on medical device-related issues are essential for the program [9]. 

The limitations of this study are a limited sample size and the study being conducted at a single tertiary care teaching institution, both of which may limit the generalizability of the findings. Additionally, as the data were based on participant perceptions, responses may be subject to recall bias.

## Conclusions

This study identified a significant gap between the recognition of device-related problems and their formal reporting within the institutional MV framework. Underreporting was shaped by limited awareness, fear of blame, and the perceived complexity of the reporting process, collectively reducing the system’s effectiveness.

Addressing these barriers requires structured sensitization initiatives, simplification of reporting procedures, and the promotion of a non-punitive culture. Timely feedback and continuous review of reported events can further reinforce reporting behavior and facilitate corrective action. Strengthening these components is essential for improving MV practices, device safety, and ultimately enhancing patient care.
